# Copy number polymorphism in plant genomes

**DOI:** 10.1007/s00122-013-2177-7

**Published:** 2013-08-29

**Authors:** Agnieszka Żmieńko, Anna Samelak, Piotr Kozłowski, Marek Figlerowicz

**Affiliations:** 1 Institute of Bioorganic Chemistry, Polish Academy of Sciences, Noskowskiego 12/14, Poznan, Poland; 2 Institute of Computing Science, Poznan University of Technology, Piotrowo 2, 60-965 Poznan, Poland

## Abstract

Copy number variants (CNVs) are genomic rearrangements resulting from gains or losses of DNA segments. Typically, the term refers to rearrangements of sequences larger than 1 kb. This type of polymorphism has recently been shown to be a key contributor to intra-species genetic variation, along with single-nucleotide polymorphisms and short insertion-deletion polymorphisms. Over the last decade, a growing number of studies have highlighted the importance of copy number variation (CNV) as a factor affecting human phenotype and individual CNVs have been linked to risks for severe diseases. In plants, the exploration of the extent and role of CNV is still just beginning. Initial genomic analyses indicate that CNVs are prevalent in plants and have greatly affected plant genome evolution. Many CNV events have been observed in outcrossing and autogamous species. CNVs are usually found on all chromosomes, with CNV hotspots interspersed with regions of very low genetic variation. Although CNV is mainly associated with intergenic regions, many CNVs encompass protein-coding genes. The collected data suggest that CNV mainly affects the members of large families of functionally redundant genes. Thus, the effects of individual CNV events on phenotype are usually modest. Nevertheless, there are many cases in which CNVs for specific genes have been linked to important traits such as flowering time, plant height and resistance to biotic and abiotic stress. Recent reports suggest that CNVs may form rapidly in response to stress.

## Introduction

For a long time, it was assumed that single-nucleotide polymorphisms (SNPs) and small insertion-deletion polymorphisms (indels) were the main types of DNA alterations underlying intra-species genomic variation. Quite recently, copy number variation (CNV) has been recognized as another common type of polymorphism in the genomes of humans, animals and plants. CNV results from unbalanced DNA modifications, which trigger changes in the number of copies of a particular DNA sequence. Typically, copy number variants (CNVs) encompass relatively large DNA segments (from 1 kb to several Mb). However, recent technical developments, especially next-generation sequencing (NGS), have enabled the identification of much shorter polymorphic regions (20–50 bp), which are traditionally defined as indels (Alkan et al. 2011).

Several mechanisms have been postulated to explain the formation of CNVs. One potential mechanism is non-allelic homologous recombination (NAHR) between DNA segments of high similarity that are not alleles. NAHR usually involves low-copy repeats (LCRs)—DNA segments larger than 1 kb that are generated during ancient duplication events. Depending on the LCR location, NAHR can lead to intrachromatid, interchromatid or interchromosomal rearrangements. The type of rearrangement depends on LCR orientation: the repeats may be direct, opposite or mixed. The orientation determines whether NAHR leads to the deletion, reciprocal duplication or inversion of the DNA segment flanked by the LCRs (Gu et al. 2008). Another potential mechanism is fork stalling and template switching (FoSTeS). FoSTeS is caused by DNA replication errors. It occurs when the replication fork stalls at one position; the nascent strand disengages from the lagging DNA template in that fork and transfers to another replication fork in close physical proximity, then re-anneals and primes DNA synthesis from that site. The template switching is driven by microhomology between the original and the invaded DNA strands. Depending on the number of switching events, the location of the invaded fork (upstream or downstream from the previously used fork) and whether the leading or lagging strand in the new fork were used as a new template, FoSTeS events may generate insertions, deletions or more complex rearrangements (Lee et al. 2007a; Zhang et al. 2009). A more detailed description of CNVs formation mechanisms can be found in the reviews of Gu et al. (2008) and Stankiewicz and Lupski (2010).

**Fig. 1:**
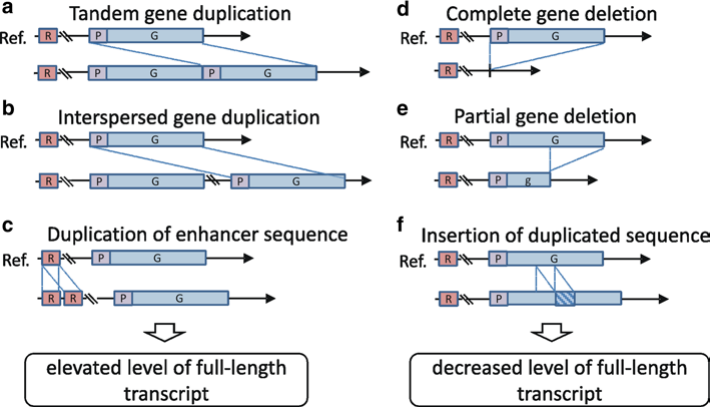
Potential effects of CNV on gene expression. **a**–**c** Examples of CNVs that result in an elevated transcript level; **d**–**f** Examples of CNVs that result in a decreased level of the full length transcript. Gene CNV (complete duplication or deletion) may change an effective gene dosage (**a**, **b**, **d**). CNV affecting an enhancer sequence may alter transcription level without change in gene copy number (**c**). Partial gene deletion (**e**) or insertion of a duplicated sequence (**f**) may disrupt gene structure and functionality. *P* promoter, *G* gene, *R* enhancer sequence

Great interest in CNVs was stimulated by the two seminal papers of Iafrate et al. (2004) and Sebat et al. (2004). Both of these papers described large-scale copy number polymorphism in the human genome. Although a few examples of CNV in specific genomic regions had been known previously, these papers initiated a research trend that led to the identification of thousands of CNVs, not only in the human genome but also in the genomes of other organisms, including plants. Currently, it is estimated that common CNVs occur in approximately 10 % of the human reference genome. Although CNVs are more common in regions almost devoid of genes (Redon et al. 2006), they are often detected in regions that contain protein-coding genes or important regulatory elements (Fig. 1). CNVs overlapping a gene may alter the expression level of the gene by virtue of changing the number of functional copies (Fig. 1a, b, d). CNVs may also affect gene regulation by position effects, as may be the case when they encompass gene regulatory sequences, even those located several Mb away (Fig. 1c). CNVs that partially overlap a gene sequence may disrupt the structure of the gene and impair its function (Fig. 1e, f).

CNVs often have phenotypic effects. In humans, variations in gene copy number have been shown to modify the risk of psoriasis (CNV affecting the β-defensin genes), HIV infection (CNV affecting the *CCL3L1* gene) and osteoporosis (CNV affecting *UGT2B17* gene) (Hollox et al. 2008; Yang et al. 2008; Gonzalez et al. 2005). In addition, CNVs are the most common somatic mutations observed in cancer genomes, primarily affecting the copy number of tumor suppressor genes and proto-oncogenes (Lee et al. 2007b; Frank et al. 2007; Shlien et al. 2008; Yoshihara et al. 2011; Stadler et al. 2012). CNVs in specific genome regions may be linked to some instances of autism, schizophrenia, epilepsy, Parkinson’s or Alzheimer’s disease (Rovelet-Lecrux et al. 2006; Weiss et al. 2008; Stefansson et al. 2008; Helbig et al. 2009; Pankratz et al. 2011; Liao et al. 2012). In addition, hundreds of rare, often de novo CNV events have been shown to significantly increase autism risk in children. A recent study showed that this disorder is associated with genome instability; global increases in both common and rare large duplications were observed in the genomes of children with autism (Girirajan et al. 2013).

In contrast, CNVs in plants have not been so thoroughly studied. It is only in the last 5 years that CNVs have attracted the attention of plant biologists and geneticists, leading to the first estimates of the extent of CNV in plant genomes. In this review, we will present current knowledge about the occurrence of CNVs in model and crop plants. We will also present examples of the association of CNVs with particular plant phenotypes. As the reader will see, the definition of CNV used in plant research is broader than in human- and animal-oriented studies, in which CNV events are attributed to individual genomes. In plant genetics, the individual organisms are mainly treated as representatives of one of the following sub-types: (1) cultivars (also named varieties), which are distinct, often intentionally bred subsets of a species that will behave uniformly and predictably when grown in the environment to which they are adapted or (2) accessions, which are collections of plant material from a particular location that are given unique identifiers (definitions after Aubry et al. 2005). This is justified by the fact that the cultivars/accessions are frequently maintained in laboratory conditions for a long time (often as inbred lines), with little influence of selective forces. In addition, many plants, including model species, are mainly self-pollinating; therefore, their genotypes are considered to be highly homozygous. Accordingly, CNVs in plants are often recognized and discussed as polymorphisms distinguishing cultivars/accessions of one species rather than affecting individual plants (Cao et al. 2011; Xu et al. 2011; Chia et al. 2012). Nevertheless, intracultivar heterogeneity is a recognized phenomenon, especially among crop plants, and some (relatively few) studies have been devoted to the exploration of genetic variation among individuals (DeBolt 2010; Ossowski et al. 2010; Haun et al. 2011).

There is, however, one more issue that needs to be highlighted in the context of CNV analysis: polyploidy. Because of whole-genome duplication events in the evolutionary history of most plant species, polyploidy is common in plants. Some of the duplicated genes may be retained as multiple copies, while other gene pairs may have diverged from each other, or some of the copies may have been lost from the homologous chromosomes. Thus, in polyploid plants, variant copy number is calculated as copies per haploid genome (Swanson-Wagner et al. 2010; Saintenac et al. 2011; Díaz et al. 2012; Cook et al. 2012), in contrast to humans and animals, in which copy number is expressed relative to the diploid genome.

## Methods of genome-scale CNVs detection

Several experimental methods are used to detect CNVs: quantitative PCR, in situ fluorescent hybridization (Weaver et al. 2010), the paralogue ratio test (Armour et al. 2007), multiplex amplifiable probe hybridization (Armour et al. 2000) and multiplex ligation-dependent probe amplification (Marcinkowska-Swojak et al. 2013). Although most of these methods allow for high-throughput genotyping of a particular variant in multiple DNA samples, they are not suitable for a genome-scale analysis and have limited use in CNVs discovery. Current experimental approaches for genome-scale CNVs discovery and genotyping are mainly based on microarrays and NGS. These methods have been recently extensively reviewed in the literature (Yau and Holmes 2008; Medvedev et al. 2009; Alkan et al. 2011). Two genome-scale methods have had the greatest impact on CNV research in plants: array-based comparative genome hybridization (CGH) and reference genome-based NGS. In the CGH approach, DNA probes are immobilized on an array, which enables simultaneous hybridization and detection of target sequences with a resolution that depends on the number and type of immobilized probes. Analysis of copy number is based on the relative amounts of signal from tested and reference genomic DNA samples binding to the probes. The reference sample in CGH analysis is usually the genomic DNA of the species (or accession) for which the microarray probes were designed. The ability of the assay to detect a particular region in the tested genome depends on its homology to the array probes. For this reason, CGH is always biased toward the detection of deletions (relative to the reference genome sequence), whereas DNA segments present in the tested genome but not in the reference remain undetected due to a lack of representative probes. Moreover, lower signal intensity observed for the tested genome may result not only from CNVs but also from other types of sequence polymorphisms that affect probe hybridization and produce a signal imbalance, resulting in false positive errors (Springer et al. 2009).

The second approach—NGS—is a high-throughput DNA sequencing technology. Modern NGS platforms, such as Illumina or ABI/SOLID, generate tens of millions of short reads in parallel (usually shorter than 100 nt) from the genomic DNA template. Signatures of CNVs in NGS data can be obtained by one of the four analytical approaches, or by a combination of them. Analysis of relative increases and decreases in sequence coverage by short reads (read-depth method) provides information about duplications and deletions. It allows for calculating the absolute copy numbers of genomic segments. Although typical analysis pipelines for NGS data involve mapping the reads to a reference genome, de novo assembly of the non-mapping reads (assembly method) allows for the discovery of new sequence variants that are not represented in the reference DNA. Additional information comes from the analysis of paired-end reads, which provide estimates of the distances between two reads and their orientation (read pair method), thereby allowing for the detection of insertions, deletions and inversions. In the case of longer reads, the exact breakpoints of all variant classes may be detected when the reads map discontinuously to the reference genome (split read method). The NGS approach has been proven effective for the discovery and mapping of structural variants at nucleotide-resolution in plants, animals and humans (Daines et al. 2009; Yoon et al. 2009; Mills et al. 2011; Cao et al. 2011; Bickhart et al. 2012). The main drawbacks of NGS are the following: difficulty with mapping short reads to DNA repeats (Treangen and Salzberg 2011) and platform-specific biases, which result in lower read coverage of some parts of the genome (for example, GC-rich regions) (Dohm et al. 2008). This type of sequencing also remains quite expensive.

## CNVs are prevalent in plant genomes

There is growing evidence that CNVs are prevalent in plant genomes (Table 1). The first plant species that has been extensively genotyped for CNVs is maize. Three important studies used CGH for CNVs detection with maize inbred line B73 as the reference genome for probe design and as the CGH reference (Springer et al. 2009; Beló et al. 2010; Swanson-Wagner et al. 2010). The first study used a high-resolution genome tiling array to detect CNVs in inbred line Mo17 (Springer et al. 2009). The two following studies were focused on gene-coding regions only and involved multiple maize lines: 13 lines in a study by Beló et al. (2010) and 19 in a study by Swanson-Wagner et al. (2010), who also assayed 14 lines of the wild maize relative, teosinte (*Z. mays* ssp. *parviglumis*). Line Mo17 was analyzed in all the above studies, making it possible to compare results across studies. Springer et al. (2009) identified approximately 400 putative polymorphic regions that were present in both the B73 and Mo17 lines, but differed in the detected hybridization signal. The CNVs with higher copy number in Mo17 were covered by highly conserved probes significantly more often than the average, and they also more often contained genes or were located near genes. Variants with higher copy number in B73 were evenly distributed across the regions represented on the microarray. This difference most likely reflects different levels of homology of the coding and intergenic regions between the genomes of the tested and reference lines. It was also observed that, although CNVs were detected on most of the maize chromosomes, they were not uniformly distributed. Several highly conserved regions that exhibited few CNVs or no CNVs were located mainly near the centromeres. This distribution pattern was later confirmed by two other CGH studies (Beló et al. 2010; Swanson-Wagner et al. 2010). All three studies also described the existence of presence–absence variants—specific CNVs where DNA regions are present in one genome but missing from the other line. Due to the CGH array design, the detected variants more often indicated decrease in copy number or complete deletion in the tested genome, relative to the B73 reference. Beló et al. (2010) reported that 57 % of all CNVs detected in their study occurred in lower copy number in the non-B73 lines. Swanson-Wagner et al. (2010) identified seven times more copy-loss events than copy gain events in the tested genomes, including presence–absence variants. One of the biggest presence–absence regions of this type, 2.6 Mb in size, located on the short arm of chromosome 6 and spanning 25 maize genes, has been shown to be missing from multiple maize inbred lines (Springer et al. 2009; Swanson-Wagner et al. 2010; Beló et al. 2010).

**Table 1:** Genome-scale CNV genotyping studies in plant genomes

Method	Accessions	CNVs count and characteristics	Gene content	References
Maize				
CGH, 2.12M NimbleGen 45–60-mer probes, matching B73 genome	Mo17 and B73 accessions	>400 CNVs and >1,700 presence–absence variants were identified (according to most stringent analysis criteria); detected differences mainly indicated lower copy number in Mo17	At least 50 genes were located in CNVs segments and 180 in presence–absence variants	Springer et al. (2009)
CGH, 105K Agilent 60-mer probes, matching 45,000 ESTs and unigenes of B73 line	14 inbred lines, including B73 reference line	>2,000 CNVs were identified; 42 % of regions were detected only in one line; 57 % changes indicated lower copy number in various accessions in comparison to B73; CNVs were distributed uniformly across chromosomes but higher CNV density was observed toward the telomeres	Due to probe design, all CNVs covered genic regions	Beló et al. (2010)
CGH, 120K NimbleGen 45–60-mer probes, matching 32,000 genes predicted in B73 genome	19 inbred maize accessions, 14 wild or inbred teosinte accessions	3,410 CNV genes had increased copy number in B73; 479 CNV genes had increased copy number in the tested accessions; CNV density resembled general genic density across the chromosomes; 86 % of structural variants was observed both in maize and in teosinte	Due to probe design, all CNVs covered genic regions; CNVs were observed in ~10 % of genes surveyed	Swanson-Wagner et al. (2010)
Whole-genome NGS, Illumina 75-bp paired-end reads, read-depth analysis, de novo assembly and annotation	Zheng58, 5003, 478, 178, Chang7-2, and Mo17 inbred lines	Only presence—absence variants were investigated; 296 genes putatively missing from one or more investigated lines were found; 570 putative novel genes were identified which were absent from B73 reference genome but present in the other of the six inbred lines; 157 genes were confirmed to be missing from B73, while about 300 are likely to be present in B73 line but not in the current genome sequence release	All analyzed presence–absence variants were in gene-coding regions; most deletion events involved only a single gene, some involved 2–4 adjacent genes, 1 large deletion on chromosome 6 of the Mo17 genome, which spans ~2 Mb involved at least 18 out of 24 genes	Lai et al. (2010)
Whole-genome NGS, Illumina 76-100 bp paired-end reads, read-depth analysis	83 maize lines, 17 *Zea mays* ssp. *parviglumis* lines, 2 *Z. mays* ssp. *Mexicana* lines, 1 *Tripsacum dactyloides* line	90 % of the non-overlapping 10-kb windows showed variation in read depth (at 1 % false discovery rate) and 70 % of windows had such variation in at least 10 of analyzed lines.	10,000 gene-coding regions (32 %) exhibited at least twofold variation in read depth	Chia et al. (2012)
Arabidopsis				
Combination of CGH (Affymetrix Tiling 1.0R arrays) and whole-genome NGS (Illumina 35-36 bp single or paired-end reads, read-depth analysis)	Eil-0, Lc-0, Sav-0, Tsu-1, Col-0 (used as a reference) accessions	55,000 25-bp tiles, on average were detected in each accession, which had relative hybridization signal ratio <−1.0 (log2) compared to the reference DNA and 0 read coverage across the entire length	1,220 (Eil-0), 1,312 (Lc-0), 1,344 (Sav-0) and 987 (Tsu-1) genes with deletions were identified, over 36 % of deletions affected coding regions and transposable element genes were over-represented; about 20 % of protein-coding gene deletions were common in the four accessions	Santuari et al. (2010)
Whole-genome NGS, Illumina 42-64 bp paired-end reads, read-depth and paired-end analysis, de novo assembly	80 naturally inbred accessions representing eight geographic regions from Eurasia and North Africa	1,059 copy number variable regions were inferred, each represented by 1–13 CNV genotypes; CNVs size ranged from 1 to 13 kb	393 CNVs overlapped with coding sequences, covering over 500 protein-coding genes	Cao et al. (2011)
Whole-genome NGS, Illumina 36–75 bp single- and paired-end reads, read-depth and paired-end analysis, reference-based assembly	Ler accession (comparative analysis to Col0)	2,315 large indels including CNVs were found in Ler, widely dispersed along chromosomes	316 genes were affected by large indels; 130 single-copy genes had complete deletion in Ler; 107 Ler-specific genes were predicted	Lu et al. (2012)
Rice				
CGH, 720K NimbleGen 45-60-mer probes, 500 bp spacing	*Oryza sativa* ssp. *japonica* (Nipponbare) and *O. sativa* ssp. *indica* (Guang-lu-ai 4)	641 CNVs covering ~7.6 Mb of the rice genome were found; CNVs ranged from 1 to 180 kb; most CNVs indicated lower copy number in Guang-lu-ai 4	500 genes with lower copy number and 19 genes with higher copy number were identified in Guang-lu-ai 4 in comparison with Nipponbare	Yu et al. (2011)
Whole-genome NGS, Illumina 45–100 bp paired-end reads, read-depth and paired-end analysis, de novo assembly	40 cultivated rice accessions (Nipponbare was used as a reference) and 10 accessions of wild *O. rufipogon* or *O. nivara*	1,415 novel genes were found (48 % of them were observed in only one accession and 22 %—only in wild rice); 1,327 possible gene loss events were detected by read-depth analysis and 839 were supported by paired-end mapping; 1,676 CNVs with increased copy number in at least one accession were found	All analyzed presence/absence variants and over 50 % of CNVs covered genic regions; 39 % of CNV genes coded for hypothetical or functional unknown proteins and many of the annotated genes were disease-resistance related	Xu et al. (2011)
Sorghum				
Whole-genome NGS, Illumina 44 bp paired-end reads, read-depth and paired-end analysis, de novo assembly	Keller, E-Tian, Ji2731 and BTx623 (used as a reference) accessions	16,487 presence/absence variants with average length of 2,394 bp were found; 17,111 CNVs (13,427 gains and 3,684 losses) of 2 kb—48 Mb were detected	Presence/absence variants co-localized with 1,416 genes; CNVs co-localized with 2,600 genes; 32 of them were identified in all three lines	Zheng et al. (2011)
Soybean				
CGH, 700K NimbleGen 50–75 bp probes with 1 kb median interval; exome NGS, NimbleGen soybean exome chip, Illumina 76-bp paired-end reads	Kingwa and Williams cultivars; individuals of Williams 82 cultivar	High level of structural variation was observed between Williams and Kingwa genotypes on all 20 chromosomes; significant level of CNV was also observed among individuals of Williams 82 cultivar, mainly within known regions of heterogeneity; most of those CNVs were also detected between the parental Williams and Kingwa genotypes	25 genes showed presence–absence variation between Williams 82 individuals; 5 of them were LRR genes; 22 of them reside within 10-Mb region of chromosome 3	Haun et al. (2011)
CGH, 700K NimbleGen array, 50-75 bp probes with 1 kb median interval; exome NGS, NimbleGen soybean exome chip; Illumina 76-bp paired-end reads	Archer, Minsor, Noir 1, Williams 82 (used as a reference) accessions	188–267 CNVs per genotype comparison were discovered, with the median size 18–23 kb; at least 133 presence–absence variants were found; unequal distribution of CNVs was observed (e.g., little variation on chromosomes 5 and 11 but extended variation regions on chromosomes 3 and 18)	672 genes localized within CNVs; they were mainly copy-loss event; genes with function in disease resistance and response to biotic stress were abundant	McHale et al. (2012)
Whole-genome NGS, Illumina 45- or 76-bp paired-end reads, read-depth analysis, paired-end mapping	17 wild and 14 cultivated accessions	Over 186,000 presence–absence variants were identified between wild and cultivated soybeans; comparison of genomes of wild W05 accession (de novo sequenced at 80×) and the reference Williams revealed over 5,500 large presence–absence variants (>500 bp)	856 genes were localized within regions of variation between W05 and Williams 82; over 40 % of them related to binding, metabolic and catalytic processes; 28 variants were absent from genomes of all cultivated accessions and were primarily related to disease resistance and metabolism	Lam et al. (2010)
Wheat				
Liquid-phase targeted exome NGS, Illumina 40-bp single end reads, read-depth analysis	Tetraploid *Triticum dicoccoides* (wild) and *T. durum* cv. Langdon (cultivated)	85 CNVs and 9 deletions were identified: 77 copy gain events/8 deletions were found in the cultivated genome and 8 copy gain events/1 deletion in the wild wheat	Genes within CNVs encoded proteins involved in response to biotic and abiotic stresses, regulating gene expression or translation, cellular metabolism and kinases	Saintenac et al. (2011)
Potato				
BAC-FISH analysis, using 18 randomly selected BAC clones mapping to potato chromosome 6	Atlantic and Katahdin cultivars; selected BACs were surveyed in additional 14 cultivars	6 BACs generated signals suggesting deletions in Atlantic and Katahdin cultivars. For BACs RH102I10 and RH83C08, deletions were detected in multiple cultivars	One BAC clone RH102I10 was analyzed in terms of gene content. It spans 19 annotated genes; 4 of them were analyzed and their normalized transcript levels correlated positively and significantly with RH102I10 copy number in different genotypes; in addition, female gametes with fewer copies of RH102I10 were found to be inferior compared with those with more copies of this CNV	Iovene et al. (2013)

Recently, the CGH method has also been applied to CNV detection in several model plant species and—similarly to the maize studies—has provided mainly examples of gene copy loss in the tested genomes. Among the 641 identified CNVs that distinguished two rice cultivars, Nipponbare (*O. sativa* ssp. *japonica*) and Guang-lu-ai 4 (*O. sativa* ssp. *indica*), the majority of CNVs indicated copy loss of genomic segments in Guang-lu-ai 4 (Yu et al. 2011). The exact rate of deletions in the Nipponbare cultivar could not be estimated, as the oligonucleotide array used in this study was designed to represent only the *japonica* cultivar. The *japonica* and *indica* subspecies diverged approximately 0.4 million years ago and display a high level of DNA sequence variation (Ma and Bennetzen 2004). CNVs identified in rice were distributed across all 12 chromosomes and comprised ~1.8 % of the rice genome. The majority of CNVs were smaller than 10 kb (67.4 % of variants) although larger CNVs were detected as well, up to a size of 180.7 kb.

In soybean, a CGH study was performed to detect CNVs in three cultivars, Archer, Minsor and Noir 1, using genomic DNA of the recently sequenced Williams 82 cultivar as a reference and as a basis for array probe design (McHale et al. 2012). Several hundred CNVs, including presence–absence variants, were detected in each of the genomes tested. The median variant size was 18–23 kb, depending on the cultivar. The CNVs in the soybean genomes had a discontinuous distribution, with very large stretches of DNA showing little or no evidence of CNV (e.g., regions covering most of chromosomes 5 and 11). As in the maize and rice CGH studies (Springer et al. 2009; Swanson-Wagner et al. 2010; Beló et al. 2010; Yu et al. 2011), the CNVs detection was biased toward copy loss in the tested cultivars.

## Large-scale population sequencing studies reveal adaptive dynamics of plant genomes

Although NGS is still an expensive method, it has proven useful for population-scale genotyping studies. In humans, a large number of individual genomes have been sequenced at low coverage to catalog CNVs and determine their frequency and distribution. This huge project comprises more than 2,300 samples, including unrelated individuals and trios (parents and a child) (Mills et al. 2011). Population-scale sequencing studies are also underway in plant genetics. They mainly aim to uncover patterns of genetic variation among cultivars/accessions and to provide a data resource for association studies. In such an approach, individual plants are assumed to be representative of the homogenous genetic pool of a particular cultivar/accession.

### Arabidopsis 1001 Genomes Project

The 1001 Genomes Project is the largest ongoing plant genome sequencing initiative. The aim of this project, which started in 2008, is to catalog the genetic variation of *Arabidopsis thaliana* (Arabidopsis) by sequencing the genomes of more than 1,000 accessions (Weigel and Mott 2009). Natural Arabidopsis accessions exhibit great variation in phenotype, including features of their morphology, metabolite profiles, germination behavior, resistance to disease, and more. Accordingly, these naturally occurring inbred lines (Arabidopsis is highly self-pollinating) are considered excellent resources for studying the putative connections between genetic variation and phenotype. Data generated within the 1001 Genomes Project are made freely available to the community, enabling analysis of various aspects of the structure of the Arabidopsis genome. Currently, data for about five hundred Arabidopsis genomes have already been released (http://www.1001genomes.org/accessions.html), and a comprehensive analysis of 80 of them has been published (Cao et al. 2011). The accessions selected for this sub-study represented six distinct geographic habitats of Arabidopsis, spanning Europe, Central Asia and North Africa. Read-depth analysis of NGS data revealed the presence of 1,059 CNVs in the Arabidopsis genome. The detected CNVs ranged from 1 to 13 kb and covered 2.2 Mb of the reference genome (approximately 2 %). For 40 % of those regions, 3–13 distinct copy number genotypes have been identified in the analyzed population. Over 85 % of those variants were detected in more than one accession. Apart from evaluation of read depth, the authors used the read pair and assembly methods to find deletions and insertions. They detected multiple deletions ranging in size from 20 bp to many kb, with approximately 5 bp precision. Finally, they managed to recover ~43,000 contigs (0.2–9 kb in size) with little (~50 bp) overlap with the reference genome (Col-0 accession). Some of those regions map to the genome of a related species, *Arabidopsis lyrata*, which suggests that their origin preceded Arabidopsis line divergence and that subsequent deletion events took place in the Col-0 accession.

### Maize Panzea Project

Another large-scale population sequencing effort, the Panzea project (http://www.panzea.org), is devoted to exploring genome architecture and variation in maize. The project mainly aims to identify the genetic background of complex traits in maize such as flowering, plant height and kernel quality, the control of which may result in future improvements in crop yield and sustainability (Canaran et al. 2008). In addition, the effect of domestication on the genome is being investigated by comparing maize and teosinte genomic data. Maize is a primarily outcrossing crop and displays tremendous phenotypic variation among the lines. Nearly 85 % of the B73 genome is annotated as transposable elements (Schnable et al. 2009). Moreover, recent SNP analysis and RNA-sequencing approaches provided evidence of great nucleotide diversity in maize cultivars (Gore et al. 2009; Hansey et al. 2012). It is estimated that approximately 30 % of the low-copy genes present in various maize inbred lines are not present in the B73 genome, which means that a substantial portion of the maize genome remains undiscovered. As explained earlier in this review, the CGH studies of maize lines, although extensive, were not devoted to discovery of this type of CNV (Springer et al. 2009; Beló et al. 2010; Swanson-Wagner et al. 2010).

In one of their recent reports, the Panzea consortium described the analysis of high-throughput sequencing data from 103 inbred maize lines, including both domesticated and wild-type lines (Chia et al. 2012). Read-depth analysis of NGS data was performed across the whole-genome sequence, using 10-kb non-overlapping windows. As much as 90 % of such regions showed at least twofold variation in read depth (at a 1 % false discovery rate), and 70 % of the windows had such variation in at least ten of the analyzed lines. Altogether, this study showed that the genetic diversity of maize cultivars is even greater than suggested by previous estimates based on CGH studies.

### Rice variation catalog

Rice is a crop of extreme agricultural importance; it is consumed in great amounts around the world. It was domesticated approximately 10,000 years ago in China, and cultivated accessions underwent substantial phenotypic changes compared to their wild ancestors. The domesticated lines can be further subdivided into several genetically distinct groups (Garris et al. 2005). As a step toward creating a comprehensive catalog of genome variation in both cultivated and wild rice, 50 accessions representing major groups of cultivated rice (ssp*. indica* and *japonica*) as well as wild rice accessions (*O. rufipogon* and *O. nivara*) were sequenced (Xu et al. 2011). Analysis of the sequencing data revealed more than 1,400 novel genes, nearly 50 % of which were found only in one accession, and over 20 % were specific to wild rice. A similar amount of possible gene loss events (more than 1,300) relative to the reference genome (“Nipponbare”, ssp. *japonica*) were detected as well, most of which corresponded to unannotated proteins. In addition to presence–absence variants, nearly 1,700 CNVs were detected, many of them (21 %) shared by at least five accessions. However, it should be noted that despite numerous re-sequencing projects in total involving hundreds of rice accessions, both domesticated and wild-type, little is known about CNVs in the rice genome. It is a natural consequence of the fact that most of these projects were focused mainly on SNP identification (Huang et al. 2010, 2012, 2013; Jeong et al. 2013). Accordingly, the analysis of the existing NGS data directed towards CNVs discovery may help to elucidate their impact on rice genome.

## CNVs commonly overlap genes

As previously demonstrated for human and animal genomes, plant CNV density correlates with repeat density and inversely correlates with gene density (Emerson et al. 2008; Conrad et al. 2010; Chia et al. 2012). Still, numerous CNVs overlap protein-coding regions. The exact number of genes overlapping CNVs in a given species varies between experiments. For example, five different experiments in maize put the number of genes in CNV-affected regions anywhere from 230 to more than 10,000 (Table 1). The factors that account for such differences between experiments include the following: the sensitivity of the method, the number of genes surveyed (in CGH, the number of genes interrogated depends on the array design, in NGS it depends on library preparation, sequencing depth and accuracy of the reference genome assembly), the analysis algorithms and statistical cut-off thresholds, and the number of samples compared. Nevertheless, many CNVs were identified in at least two of the five studies. For example, high concurrence between presence–absence variations identified in Mo17 genome was observed for the CGH and NGS data (Springer et al. 2009; Lai et al. 2010). In addition, genes identified as copy number variable in multiple lines in another CGH study also showed high average variation in the NGS-based analysis (Swanson-Wagner et al. 2010; Chia et al. 2012). The latter study estimated that 32 % of the genes annotated in the B73 reference genome are affected by CNVs. This is much greater than the proportion of genes affected in Arabidopsis, rice or soybean (see Table 1), and it correlates with the overall higher genetic diversity and spectacular phenotypic diversity of maize. The large number of genes overlapping the discovered CNVs may also explain, at least in part, the transcriptome variation observed among different maize lines. The semi-quantitative analysis of NGS-transcriptomic data revealed that multiple transcripts differed in abundance among 21 inbred maize lines (possible classification groups were: “no”, “low”, “medium” or “high” expression level) (Hansey et al. 2012). In addition, many transcripts annotated in the reference genome were absent from specific lines, and in other cases, novel transcripts were found in specific lines. Swanson-Wagner et al. (2010) observed that 86 % of identified CNVs are present both in wild-type and domesticated lines, suggesting that the majority of the observed gene involving CNV events in maize preceded domestication and that they are not the product of artificial selection. Rare CNVs (i.e., those unique to a single line) were observed frequently in teosinte. Because 10 of the 14 teosinte lines used in the cited study were segregating, the authors concluded that many naturally occurring CNVs covering gene-coding regions may be non-neutral and may, therefore, be tolerated only in the heterozygous state, whereas breeding eliminates those CNVs from the genomes of highly inbred lines. On the other hand, in the study by Beló et al. (2010), the rate of occurrence of particular gene CNVs only in a single domesticated line was calculated to be much higher—about half of all CNVs observed. Although different maize lines were genotyped in the two experiments (except for Mo17 and B73), those discrepancies point to the need for more in-depth analysis of genomic data to evaluate the range of CNVs occurrence in maize (and other plants) as well as the rate of maize evolution.

### NB and RLK multigene families are especially prone to CNV

According to population sequencing studies, a major fraction of genes located within CNV regions code for hypothetical or unknown proteins (Xu et al. 2011; Cao et al. 2011). Among the functionally annotated genes, those which are usually overrepresented within CNV regions are genes encoding proteins with a nucleotide binding domain (NB) and one or more leucine-rich repeat (LRR) domains (known as NB-LRR genes), as well as genes encoding receptor-like kinases (RLK). Both NB-LRR and RLK genes constitute large gene families, and many of them are functionally classified as defense-related. Not surprisingly, GO term enrichment analysis of the 672 genes located within CNV regions in soybean revealed that genes related to disease resistance and biotic stress response were significantly overrepresented (McHale et al. 2012). Similar observations have been made for Arabidopsis and rice, where disease resistance genes represent a significant fraction of genes in CNV regions (Xu et al. 2011; Cao et al. 2011; Lu et al. 2012). High levels of duplication ensure the variability of defense genes, and such variation is advantageous in the face of changing environmental conditions. Indeed, those genes seem to be under weaker purifying selection or under stronger diversifying selection than other duplicated genes, such as genes involved in protein translation (Korbel et al. 2008; Warren et al. 2010; Lu et al. 2012). The genes of the NB-LRR family represent the largest class of resistance (R) genes that are involved in race-specific recognition of pathogen avirulence determinants. R genes are subject to strong selective pressure promoting coevolution with pathogen effector proteins. Depending on the presence or absence of particular pathogens, the pressure for the selection of corresponding R genes dramatically changes, leading to rapid evolution (Guo et al. 2011; McHale et al. 2012; Ashfield et al. 2012; Luo et al. 2012).

CNVs were reported to overlap multigene families more often than unique genes in many plant species (Swanson-Wagner et al. 2010; Cao et al. 2011; Xu et al. 2011; Zheng et al. 2011; Chia et al. 2012; McHale et al. 2012). Recent GO term enrichment analysis of CNVs identified by CGH in soybean suggested, however, that higher CNV frequency correlates specifically with the NB and RLK gene families, not with large gene families in general. When those genes were removed from the GO term enrichment analysis of CNVs, the frequency of large gene families overlapping CNV regions did not differ much from the overall frequency of genes within those regions (McHale et al. 2012). This suggests that the large size of a gene family is not sufficient to promote CNVs formation and that some families are more affected by copy number polymorphism than others. In addition, gene members of a given family are not equally predisposed to CNV. Genes localized in clusters, especially in tandem arrays, seem to undergo copy number changes more often than isolated family members (McHale et al. 2012), which is consistent with recombination-based mechanisms of CNV formation, although it does not exclude alternative mechanisms.

## Associations of CNVs with plant phenotypes

Despite the prevalence of CNVs in plant genomes and their frequent overlap with protein-coding regions, only a few have been associated with particular phenotypes on the morphological, physiological or developmental level. Paralogous plant genes are often functionally redundant. Therefore, variations in copy number of one member of a gene family may trigger quantitative rather than qualitative changes, making the CNV-trait association difficult to detect. Still, a growing number of reports provide evidence that copy number polymorphisms contribute to natural genetic variation and control important adaptive traits in plants (Table 2).

**Table 2:** Confirmed examples of CNV affecting plant phenotype

CNV region	Attribute	Gene(s)/product(s)	Description	References
Soybean				
Rhg1 locus on chromosome 18, 31 kb	*rhg1*-*b* allele-associated resistance to *Heterodera glycines* nematode	*Glyma18g02580*/amino acid transporter, *Glyma18g02590*/α-SNAP protein, *Glyma18g02610*/wound-inducible domain containing protein	Overexpression of all three genes together (but not individual genes) provides resistance to nematode; 10 tandem copies are present in *rhg1*-*b* haplotype while only 1 copy is present in susceptible haplotype	Cook et al. (2012)
Palmer amaranth				
Distributed all over the genome	Acquired resistance to glyphosate treatment	*EPSPS*/5-enolpyruvylshikimate -3-phosphate synthase	Increased copy number of *ESPS* gene triggers glyphosate resistance (40 -100 times more copies in resistant plants in comparison to susceptible plants); *EPSPS* gene copy number correlates with transcript and protein levels as well as with a herbicide dose survival rate	Gaines et al. (2010, 2011)
Barley				
Boron-tolerance QTL on chromosome 4H	High boron tolerance of Algerian landrace Sahara 3771	*Bot1*/boron efflux carrier	Tolerant Sahara 3771 genotype contains ~4 times more *Bot1* copies (with 2 amino acid changes) and highly elevated *Bot1* transcript levels in comparison to susceptible Clipper genotype; overexpression of *Bot1* conferred boron-tolerance in yeasts	Sutton et al. (2007)
Frost resistance-2 locus on chromosome 5, genetically linked with Vrn1-locus	vrn-H1 winter allele associated with winter-hardy genotypes and Vrn-H1 spring allele associated with non-winter-hardy genotypes	A cluster of *CBF* genes/C-repeat DNA binding transcriptional activators	Tandem segmental duplications through the CBF2A–CBF4B genomic region differentiate freeze-tolerant genotypes from sensitive genotypes which carry single copies of those genes	Knox et al. (2010)
Wheat				
Vrn-1 locus on chromosome 5A	Differing vernalization-requirements associated with Vrn1-A allele, which influence flowering time	*Vrn*-*1 A/*MADS-box transcription factor	Copy number of *Vrn1*-*A* inversely correlates with vernalization requirement and flowering time (1 haploid copy in early flowering plants, 3 copies in late flowering plants and 2 copies in plants with medium phenotypes)	Díaz et al. (2012)
Ppd-1 locus on chromosome 2B	Day-neutral phenotype associated with Ppd-B1a alleles in several varieties, influencing flowering time	*Ppd*-*B1*/family member of pseudo response regulators (PRR) with a CCT domain	Day-neutral genotypes carry 2-4 haploid copies of *Ppd*-*B1* gene, while photoperiod sensitive genotype—only 1	Díaz et al. (2012)
Rht-D1 locus on chromosome 4D	Dominant *Rht*-*D1c* allele determining extreme dwarf phenotype in Aibian 1 line	*Rht*-*D1b*/ineffective DELLA protein, truncated in the region responsible for gibberellic acid response	Tandem segmental duplication (TSD) of a >1 Mb region result in two copies of the Rht-D1b; Rht1-D1c is threefold more effective in reducing plant height than a single Rht-D1b	Pearce et al. (2011), Li et al. (2012)
Rice				
Submergence 1 (Sub1) locus on chromosome 9	Tolerance-specific allele Sub1A-1 associated with enhanced submergence tolerance in *O sativa indica cultivar* FR13A	*SUB1*/APETALA2/ethylene response factor	Presence of *SUB1A* gene in submergence-tolerant accessions restrains elongation growth, economizing carbohydrate reserves to enable development of new leaves upon desubmergence; the gene is absent from all *O. sativa japonica* and most *O. sativa indica* accessions	Xu et al. (2006)
Maize				
Aluminum (Al) tolerance QTL in telomeric region of chromosome 6	Al tolerance associated with ZmMATE1 gene in a tolerant line Al237	*MATE1*/anion transporter from the MATE family; mediates root citrate efflux in response to Al	Tandem triplication of *MATE1* gene provides higher gene expression and superior aluminum tolerance in maize Al237 line, in comparison to Al-sensitive L53 line; the triplicated gene copies are 100 % identical; two other lines with amplification of *MATE1* gene (Il677a and C100-6) which also show Al tolerance share the same geographical origin as Al237 line—acidic soils of the South African tropics	Maron et al. (2013)
Tunicate1 (Tu1) locus on long arm of chromosome 4	A dominant mutation causing pleiotropic phenotype; it affects phase transition, branch meristem formation, spikelet initiation, and sex determination; predominant feature is tunicate phenotype—mature kernels of the cob are covered by glumes	*ZMM19 MADS*-*box* transcription factor	In pod corn 5′ regulatory region of ZMM19 gene is fused by a 1.8-Mb chromosomal inversion to the 3′ region of a gene expressed in the inflorescence, which leads to mild half-tunicate phenotype. A 30-kb tandem duplication of the rearranged region results in severe tunicate phenotype observed in some plants	Han et al. (2012), Wingen et al. (2012)

**Fig. 2:**
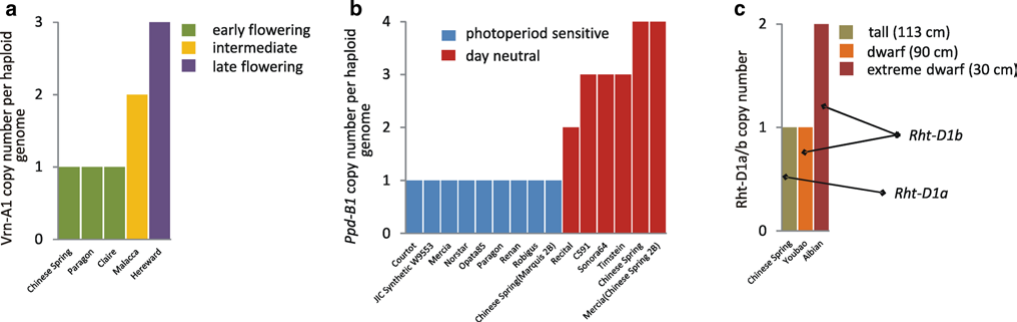
Gene CNV contributes to wheat phenotypic diversity. **a** CNV of *Vrn*-*A1* gene controls flowering time by affecting vernalization requirement; **b** CNV of *Ppd*-*B1* controls flowering time by affecting photoperiod sensitivity; **c** CNV of *Rht*-*D1b* gene (a truncated version of *Rht*-*D1a*) determines severity of plant dwarfism phenotype. In all three cases, the impact of gene copy number on observed phenotype has been verified experimentally. Source data: **a**, **b** Díaz et al. (2012); **c** Li et al. (2012)

A good example of a CNV affecting phenotype is found in the diversity of flowering times and plant heights in wheat (Fig. 2). CNVs for the genes *Vrn*-*A1* and *Ppd*-B1 were shown to contribute to differences in flowering time between the wheat varieties (Díaz et al. 2012). Plants with an increased copy number of *Vrn*-*A1*, which encodes a MADS-box transcription factor, require prolonged vernalization and exhibit intermediate or late flowering phenotypes (depending on the exact number of gene copies, see Fig. 2a). The other gene, *Ppd*-*B1*, belongs to a family of pseudo response regulators (PRR) and it has been shown to control photoperiod sensitivity in wheat. Wheat cultivars with only one copy of *Ppd*-*B1* per haploid genome are photoperiod sensitive, whereas those with increased copy number (2–4 copies), exhibit an early flowering, day-neutral phenotype (Fig. 2b). Also in wheat, a CNV has been found to determine the extreme dwarf phenotype observed in the Aibian 1 line (Li et al. 2012). In this line, tandem segmental duplication of a greater than 1 Mb region resulted in two copies of the *Rht*-*D1b* gene in the haploid genome. *Rht*-*D1b* codes for a truncated DELLA protein, lacking the gibberellic acid response region. The *Rht*-*D1b* allele itself triggers plants’ insensitivity to gibberellic acid and causes a 20 % height reduction (~90 cm in Youbao line, compared to ~113 cm in the Chinese Spring line, which is a tall wheat carrying a wild-type allele *Rht*-*D1a*). In Aibian 1 line, however, the presence of two copies of *Rht*-*D1b* results in a greater than 70 % reduction in plant height (~30 cm) (Fig. 2c).

Several confirmed examples of a CNV link to phenotype concern plant stress tolerance (Table 2). CNV of *Bot1*, a boron efflux carrier gene, has been shown to play a significant role in conferring boron tolerance in barley (Sutton et al. 2007). Another report links CNV of three soybean genes with the development of nematode resistance. In this plant, the poorly characterized *Rhg1* locus on chromosome 18 has long been known to contribute to soybean resistance to *Heterodera glycines* (soybean cyst nematode, SCN). Recent work by Cook et al. (2012) brought evidence that SCN resistance triggered by the *rhg1*-*b* allele results from *simultaneous* overexpression of three clustered but non-homologous genes: an amino acid transporter, an α-SNAP protein and a wound-inducible domain containing protein. It seems that products of those genes act in concert to convey the resistance phenotype, although the biochemical mechanism of their cooperation remains unknown. Though SCN-susceptible soybean varieties contain only one copy of each gene, resistant lines carrying the *rhg1*-*b* allele possess up to 10 tandem copies of the gene cluster. This discovery may have direct economic impact on soybean production by enabling the selection of SCN-resistant varieties based on copy number evaluation of the *Rhg1* region.

## The pace of CNVs evolution

Recently, Lu et al. (2012) provided direct insight into the rate of structural alterations introduced during a single round of meiosis in the Arabidopsis genome. Using the Arabidopsis *qrt1* mutant (a *Col/Ler* F1 hybrid), the authors produced four attached pollen grains from all four meiotic tetrads. Their progeny was obtained by pollinating a single pistil of an emasculated *Col* flower, and the genomes of the resulting plants were sequenced and analyzed. There were 21 and 32 CNVs generated by meiotic events in the two “tetrad progeny” sets. The main cause of the CNV seemed to be NAHR-mediated reshuffling of existing highly similar sequences that map to different locations in the genome. Given that meiosis can rapidly generate CNVs among siblings (as the study of Lu et al. shows), it can be concluded that de novo CNVs are frequent in plant genomes, although the majority of them most likely do not become fixed because of strong purifying selection.

**Fig. 3:**
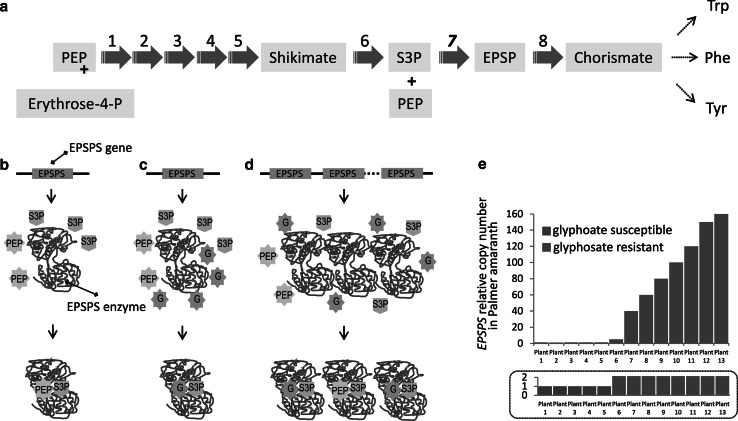
Glyphosate resistance in Palmer amaranth mediated by CNV of EPSPS gene. **a** Graphical representation of the shikimate pathway. Step 7 is catalyzed by EPSPS enzyme; **b**–**d** mechanism of EPSPS inhibition by glyphosate and its overcoming by increased number of EPSPS gene copies. In absence of glyphosate, PEP and S3P bind to EPSPS (**b**). When glyphosate is present, it competitively binds to EPSPS, mimicking an intermediate state of the ternary enzyme–substrates complex and inhibiting EPSPS (**c**). Amplification of EPSPS gene leads to production of additional protein molecules and PEP binding, even in presence of glyphosate (**d**). **e** Differences in EPSPS gene copy number between glyphosate susceptible and glyphosate-resistant Palmer amaranth individuals. *EPSPS* 5-enolpyruvylshikimate-3-phosphate synthase, *PEP* phosphoenol pyruvate, *S3P* shikimate-3-phosphate, *EPSP* 5-enolpyruvylshikimate 3-phosphate, *G* glyphosate

Changes in gene copy number may provide a way to rapidly alter the effective dosage of a gene, which directly affects phenotype to a variable extent. As long as the new variant is beneficial and has high selective pressure over many generations, the copy number alterations in a particular region may accumulate, and the phenotypic effects may intensify. A remarkable example of extremely fast evolution in a plant genome has been presented recently, and the case involves resistance to glyphosate in Palmer amaranth (*Amaranthus palmeri*)—a major weed pest in the southern part of the United States. Glyphosate is a non-selective herbicide that inhibits the activity of 5-enolpyruvylshikimate-3-phosphate synthase (EPSPS), an important enzyme of the shikimate pathway in plants (Fig. 3). The shikimate pathway leads to the conversion of phosphoenol pyruvate (PEP) to chorismate—a common precursor in the biosynthesis of aromatic amino acids (Fig. 3a). The EPSPS enzyme catalyzes the biosynthesis of 5-enolpyruvylshikimate-3-phosphate (EPSP) from shikimate-3-phosphate (S3P) and PEP (Herrmann 1995) (Fig. 3b). In susceptible plants, glyphosate occupies the PEP-binding site in the EPSPS protein, acting as a competitive inhibitor of its enzymatic activity (Schönbrunn et al. 2001) (Fig. 3c). For years, glyphosate has been successfully used to control the expansion and growth of weeds, including Palmer amaranth. About 8 years ago, glyphosate-resistant populations of Palmer amaranth were detected in Georgia, and the infested area in that state and other US states has dramatically increased since then (Culpepper et al. 2006; Gaines et al. 2010). It has been shown that Palmer amaranth resistance to glyphosate is driven by an increase in EPSPS gene copy number, which is associated with increased EPSPS transcript and protein levels as well as increased glyphosate dose survival rate (Gaines et al. 2010, 2011). Resistant plants carry an increased number of EPSPS gene copies (typically between 40–100 times more than susceptible plants). The higher production of EPSPS enzyme due to the increased gene copy number enables those plants to overcome the inhibitory effect of glyphosate, most likely by providing enough enzyme molecules to bind the physiological substrate PEP, even in presence of glyphosate (Fig. 3d, e).

The EPSPS gene CNV is not unique to Palmer amaranth. Recent and rapid increases in glyphosate resistance in common waterhemp (*Amaranthus tuberculatus*) and Arkansas populations of Italian ryegrass (*Lolium perenne* ssp. *multiflorum*) have been attributed to increased copy number of EPSPS in those plants as well (Tranel et al. 2011; Salas et al. 2012). The EPSPS example shows that the accumulation of copy number changes may serve as a mechanism of extremely rapid evolution under high selection pressure. In the case of Palmer amaranth, the random distribution of EPSPS gene copies in the genome (as observed by FISH experiments) suggests the involvement of transposable elements in the creation of new EPSPS gene copies (Gaines et al. 2010). Mobile genetic elements might have been induced and/or supported by the very strong selective pressure resulting from extensive glyphosate treatment, leading to duplication and transfer of a nearby EPSPS gene.

## Outlook

Recent genome-scale studies indicate that CNV significantly contributes to natural variation of plants. Accordingly, one can expect that CNVs play significant roles in plant evolution and adaptation. There is no doubt that the research on CNV phenomenon in plant is still at its beginning but we envision its dynamic development in the nearest future. Highly inbred and genetically homogenous plant cultivars seem to be perfect models for studying general and plant-specific aspects of CNV. This is especially true for Arabidopsis, a self-compatible plant with a small genome and a plenty of genetic tools, such as RILs (recombinant inbred lines) and MAGIC (multiple advanced generation intercross) already available (Weigel 2012).

We expect a growing approbation of CNV’s impact on plant phenotype, both in the aspect of long-term evolution as well as a mechanism of rapid adaptation to environmental challenges. Crops, which underwent fast phenotypic transformation under strong selective pressure related to domestication, may be excellent models for studying the general role of CNV in adaptation. This problem seems to be especially interesting in the context of recent reports suggesting that rapid copy number expansion of genes involved in resistance to herbicides or drugs, may take place (reviewed in Kondrashov 2012). In many such cases, CNV affected the same key genes independently in various populations or even independently in different species (Triglia et al. 1991; Widholm et al. 2001; Labbé et al. 2007; Gaines et al. 2010; Tranel et al. 2011; Salas et al. 2012). In addition, recurrent gene deletions have been observed in plants and animals, highlighting the role of presence–absence variation in rapid adaptive evolution (McGrath et al. 2011; Olsen and Wendel 2013). Those examples allow to hypothesize that CNV phenomenon may be successfully employed for directional plant improvement.

Links between CNVs and phenotypic variation also suggest that CNVs can be utilized in genome-wide association studies (GWAS), which are now based mostly on SNPs (Atwell et al. 2010). Indeed, association analysis of 5 traits involved in leaf development and disease resistance in 103 maize lines using both SNPs and CNVs revealed that CNVs contribute greatly to the variation of analyzed phenotypes and provide complementary information to SNPs (Chia et al. 2012). However, to enable the use of SNP and CNV markers by the community, integrated plant genomic variant catalogs, similar to the human Database of Genomic Variants, are needed. Increasing accessibility of NGS techniques makes such databases likely to be created in the nearest future. Still, the main limitation of NGS-based CNV discovery is lack of well-established pipelines for data analysis and imperfection of the current software to correct for technical bias in the sequence data. There are observations (including our own unpublished results) that utilizing different software for read mapping and/or CNV calling from the same sequence data, results in lists of variants which have little overlap with each other (Alkan et al. 2011). Thus, to confirm the accuracy of genome-scale CNV discovery from NGS data, variant calling should be routinely followed by experimental verification of a large fraction of inferred CNVs using one or more molecular genotyping assays (Cantsilieris et al. 2012). Currently, this process is usually limited to relatively easy verification of presence–absence variants.
